# Measuring coverage of essential maternal and newborn care interventions: An unfinished agenda

**DOI:** 10.7189/jogh.07.020101

**Published:** 2017-12

**Authors:** Liliana Carvajal–Aguirre, Lara ME Vaz, Kavita Singh, Deborah Sitrin, Allisyn C Moran, Shane M Khan, Agbessi Amouzou

**Affiliations:** 1 Data and Analytics, Division of Data, Research and Policy, UNICEF, New York, New York, USA; 2 Saving Newborn Lives, Save the Children, Washington, D.C., USA; 3 MEASURE Evaluation/Carolina Population Center, University of North Carolina at Chapel Hill, Chapel Hill, North Carolina, USA; 4 Department of Maternal and Child Health, Gillings School of Global Public Health, Chapel Hill, North Carolina, USA; 5 Jhpiego, Baltimore, Maryland, USA; 6 Department of Maternal, Newborn, Child and Adolescent Health, World Health Organization, Geneva Switzerland; 7 Department of International Health, Johns Hopkins Bloomberg School of Public Health, Baltimore, Maryland, USA

Over the past few decades, the agenda for newborn health has shifted remarkably, taking newborns from being nearly invisible in the global health agenda of 1990s to being central in discussions today. Despite this change, the decline in neonatal mortality from 1990 to 2016 has been slower than that of post–neonatal under–five mortality: 49% compared with 62% globally [1]. Newborn deaths represent 46% of all under–five deaths–of the 5.6 million under–5 deaths in 2015, nearly 2.6 million deaths occurred in the neonatal period, with a large proportion dying within the first week following birth [1,2]. Preterm birth complications (35%), intrapartum–related events (24%) and sepsis (15%) – most of which are preventable–have been identified as leading causes of neonatal deaths [3]. Although maternal mortality was estimated by the UN inter–agency group to have declined by 44% between 1990 and 2015, the reduction was far below the 75% MDG target. Approximately 303 000 women die each year from complications of pregnancy and childbirth, with 99% of deaths in low– and middle–income countries, making maternal mortality one of the indicators with the largest disparity between rich and poor countries [4]. With the majority of maternal and newborn deaths occurring around the time of birth, quality and equitable maternal and newborn care are essential to improve survival. Several global partnerships and initiatives such as the United Nations Every Woman Every Child movement (EWEC) and Every Newborn Action Plan (ENAP) have called for more focused attention on newborn health in order to end preventable newborn and child deaths [5,6]. The 2030 agenda of Sustainable Development Goals (SDG) and accompanying Global Strategy for Women’s Children’s and Adolescents’ Health (2016–2030) include a specific target for all countries to reduce neonatal mortality to at least as low as 12 per 1000 live births, further reinforcing and strengthening commitment to neonatal survival [7].

Available research and evidence on newborn health clearly highlight impending challenges and strategies to improve newborn survival. The 2013 PLOS Medicine collection on “Measuring Coverage of MNCH” and the 2014 Lancet Every Newborn Series noted gaps in the availability of metrics and data on newborn care. Additionally, the globally agreed upon monitoring frameworks as ENAP, Ending Preventable Maternal Mortality (EPMM), the Global Strategy for Women’s, Children’s and Health (2016–2030) and Countdown to 2030 – have all identified critical areas where further indicator development and data collection are needed and have begun work to test or validate indicators [8]. There is also increased recognition of the role of data in measuring progress toward the promise of an equitable future in the SDG era. This has resulted in an explicit SDG target to support countries to increase the availability of high–quality, timely and disaggregated data, including data related to newborn health.

To date, large–scale household surveys such as the UNICEF–supported Multiple Indicator Cluster Surveys (MICS) and the USAID–supported Demographic and Health Surveys (DHS) are the primary sources of population–level coverage estimates of newborn health interventions [9,10]. Household surveys have been extremely important for national and sub–national monitoring of key indicators and are invaluable as a public source of data for examining sub–national inequalities and understanding coverage gaps in intervention as well as for research purposes. However, studies have indicated that the validity of coverage measures from household surveys can vary across indicators [11–13]. Household survey programs work constantly on revisiting and refining s approaches to data collection. Following the 2013 recommendation of the Newborn Indicators Technical Working Group, some new indicators to measure care in the immediate newborn period have been added by the two household survey programmes. In addition, newborn–care related content is now also included and measured through health facility assessments.

With the increasing focus on the need for data on newborns, and availability of new data, it is time to understand these data and take stock of the findings but also of gaps. In the current context in which newborn survival is central to the global health agenda, there is an urgent need to strengthen the collection of data on newborn care, particularly on aspects related to quality of care and to identify remaining gaps as well as ensure the data are aligned with global and national monitoring needs. Attuned to this context, the series of papers in this collection provide program and policy findings on measurement of maternal and newborn care and outcomes, with implicaitons for future measurement implementation and research. The supplement provides an analysis and description of the associations and patterns of coverage and quality of recommended maternal and newborn care practices and interventions as captured at the population and facility level. It further strengthens evidence of limitation of current coverage indicators and the need for effective coverage measurement that incorporates quality of care provided. Several papers in the supplement highlight the scope of facility level data in assessing readiness to provide newborn care. Finally, the supplement assesses gaps and quality of available data on newborn health and measurement approaches across measurement platforms.

## MEASURING PROGRESS AND CHALLENGES IN NEWBORN HEALTH

### Newborn health–related measurement (data and metrics)

Improving measurement of newborn health is at the core of this supplement. Though the quality, frequency and visibility of data for newborn health have improved notably compared to a decade ago [14], gaps in availability and quality of data on newborns remain. To accelerate and monitor progress towards the global target of reducing neonatal mortality, a set of core indicators has been proposed and incorporated in several monitoring frameworks. Some core indicators like, skilled birth attendants and exclusive and early initiation of breastfeeding have been established and reported on for decades through data collected in MICS, DHS and other household surveys. As a result, nearly 75% of the countries have data available for these indicators [8,15]. On the other hand, some indicators used for global reporting, such as “postnatal care for mothers and newborns,” have been agreed upon more recently, with essential care indicators such as “thermal care” recommended for data collection in household surveys only in 2013. As highlighted by Sitrin et al in this supplement, only twelve national surveys between 2005 and 2014 included at least one indicator for immediate newborn care in addition to breastfeeding [16]. The supplement includes a series of papers addressing gaps and assessing the quality of many of these core indicators. Main findings are described below (**Table 1**).

**Table 1:** Data on newborn indicators across current global monitoring frameworks and assessed in the current collection

Newborn health–related indicators	Gaps (as identified in the current series)	Recommendation (based on studies in the series)
Content of antenatal care	Large gap between contacts and content of antenatal care	Coverage indicators should include some elements of content of care to identify true effectiveness of maternal and child health interventions.
Skilled attendant at birth	Skilled attendants even in health facilities may not be equipped to save newborns	Need to supplement and link coverage data with health facility level data and quality of care indicators.
Postnatal care for mothers and babies	Difference in data collection tools eg, questionnaires and the methodology adopted to measure PNC across survey programs has created comparability issues in coverage levels	Need to harmonize data collection tools across survey programs. Need to determine differences in coverage by individual, household, regional characteristics. Individual characteristics should include delivery–related factors.
Essential newborn care with early initiation of breastfeeding as tracer indicator: • immediate and thorough drying; • immediate skin–to–skin contact; •delayed cord clamping; • early initiation of BF	• Early initiation of breastfeeding is not a high performing tracer indicator of essential newborn care, •Coverage of skin to skin and thermal care is low	Need to collect data on newborn care practices other than breastfeeding initiation through standardized questions in household level surveys.
Service readiness for newborn care in facilities	Lack of qualified staff	Improve training and increase capacity of staff across health sectors. Increase availability of essential commodities

Postnatal care (PNC) is an important strategy to improve newborn survival. Some issues related to measurement of postnatal contacts were mentioned in the PLOS One series, “Measuring Coverage of MNCH”; it also described a few changes made to MICS and DHS questionnaires, in an attempt to address issues revealed through formative research on indicator for postnatal care. However, there has not been a systematic assessment comparing the measurement approaches implemented by MICS and DHS, the two largest sources of population–based MNCH coverage data in low and middle–income countries, which left open the question of how questionnaire differences may affect the comparability and interpretation of PNC coverage across surveys and countries. The study comparing measurement of postnatal care across the two survey programs in this supplement reveals a difference in the way questions on postnatal care for mothers and newborns are framed in MICS and DHS. MICS and DHS surveys have also followed different methodological approaches to compute the global indicator of postnatal contacts for mothers and newborns within two days following delivery, resulting in comparability issues in coverage levels across the two programs. As the evidence shows, this has implications for accurate measurement of coverage of postnatal care [17]. With an increased focus on quality of care provided, content of postnatal care may provide more helpful monitoring information to track reduction of neonatal mortality in the future.

The Every Newborn Action Plan proposed several indicators to track impact, coverage and equity of newborn health–related interventions. It proposed early breastfeeding as a tracer for essential newborn care, due to the data availability and evidence of benefits of breastfeeding. A methodological paper in this series assessed the correlation between early breastfeeding initiation and other newborn care practices [16]. The analysis found that breastfeeding initiation is not a good tracer indicator for newborn care practices and recommends improved methodologies for accurate measurement of these practices.

The quality of newborn health interventions is a significant gap that is not currently being addressed by the globally agreed upon coverage indicators to assess newborn health. It is increasingly recognized that global measures of coverage of maternal and newborn health capture main contacts with the health system but provide little information about the quality of care received. In this supplement, we assessed the gap between contact and content –as a proxy for quality–of maternal and newborn health services in 20 sub–Saharan countries and found that the gap between contact and content is excessively large in all [18].

### Newborn health policy and program

Over the past several years, there have been major advances in agenda setting for newborn health, including implementation of several globally endorsed action plans and monitoring frameworks. There has been a notable increase in the number of publications focused on newborn health, and evidence is now available for interventions that address the three main causes of newborn deaths. Recent research indicates that increased coverage and quality of preconception, antenatal, intrapartum, and postnatal interventions by 2025 has the potential to avert 71% of neonatal deaths (1.9 million, range 1.6–2.1 million) [19]. A study in this supplement analyzed the recently available data on newborn care practices and found very low coverage of skin–to–skin contact despite its protective effects against neonatal morbidity and mortality [20]. Singh et al. examined the role of individual and health system characteristics on receipt of postnatal care and found coverage to be low in Bangladesh, particularly for newborns of mothers who delivered at home and who did not report a complication. Such analysis result in better identification of the most vulnerable newborns and provide valuable programmatic insights to improve coverage [21].

Quality of newborn health interventions emerged as a key missed opportunity to accelerate newborn survival in three studies that analyze survey data from 20 sub–Saharan countries, Bangladesh and Haiti [22–24]. Two studies using health facility data assessed the service readiness to deliver life-saving newborn interventions and found that health facilities are not yet equipped to save newborns at risk of dying [23,24]. An assessment of health service environment in Malawi revealed that newborns in districts with high service readiness have higher odds of receiving essential newborn care. This study highlights that there is an urgent need to increase the level of service readiness across all facilities and in particular, the quality and training of the staff, so that all newborns–irrespective of the health facility, district or region of delivery–are able to receive all recommended essential interventions.

## RECOMMENDATIONS – CALL TO ACTION

Poor quality in newborn care is a major barrier to newborn survival, and we strongly recommend strengthening measurement of elements of content of care to improve the measurement of the coverage of maternal, neonatal and child health care. Recently, the World Health Organization and the Lancet quality improvement commission have proposed standards of care and measures assessing quality of maternal and newborn health care [25,26]. We propose that linking household survey data on coverage of interventions with facility–level data on service availability and readiness could help better measure effective coverage and identify its determinants and barriers. 

It is encouraging to note that newborn health measurement is now central to many global initiatives, and new indicators are being added to household surveys and facility assessments. However, to track progress over time and make comparisons between countries, there is an urgent need to harmonize data collection across household surveys and facility assessments. To assess whether newborns are receiving life–saving interventions, the existing standardized questions regarding newborn care practices such as thermal care and skin–to–skin contact need to be consistently added to national household surveys. The DHS now includes an optional newborn module which outlines standardized questions on newborn care, which could be added to DHS surveys. MICS also includes standard questions. However, there is some evidence of poor validity of household survey indicators especially related to timing or sequence of events around the time of birth or questions which are composite of several events such as breastfeeding within one hour of birth, newborn dried and placed on mother’s skin. For instance, these studies confirm that many indicators of intrapartum care and associated morbidities have generally low validity and reliability when assessed by women’s reports. However, some salient indicators are reported with acceptable accuracy, most notably skilled attendance at birth and cesarean section. [12,27]. Other strategies must be developed for those indicators with low validity and reliability and caution must be taken when interpreting results. The limitations of these data sources to capture the complexity of maternal and newborn health care are well documented, but in the era of the Sustainable Development Goals the importance of these sources for global tracking, monitoring national trends and understanding equity perspectives are as important as ever. Newborns also require data that can inform the decisions of more local health system actors. 

At the district level a manager who wants to optimize the health system can use national survey data to benchmark indicators at the regional level once every three to five years. But to know which inputs and health worker processes are optimized in the district, where actions are needed, and crucially whether outcomes improve as a result, different data platforms are required. The Health Management Information System (HMIS) and a well-functioning civil registration and vital statistics system have the potential to support this need and innovations to summarize and visualize these data so fit for district-level management could play an important role. Currently, UNICEF, WHO, and UNFPA are developing a standardized list of indicators for maternal and newborn health that can be consistently tracked and reported through HMIS and DHIS2. Other projects, such as the Maternal and Child Survival Program and the Quality, Equity and Dignity Network are testing and implementing these indicators, with a focus on using data for decision making at all levels. Data from HMIS will be crucial for monitoring progress toward national and global targets.
